# Supplementary material for: ANXA11 biomolecular condensates facilitate protein-lipid phase coupling on lysosomal membranes
Source: Nat Commun. 2025 Mar 21;16:2814. doi: 10.1038/s41467-025-58142-5 (PMC11928461; doi:10.1038/s41467-025-58142-5)
Supplement: Supplementary file 1 — Supplementary Information [file 41467_2025_58142_MOESM1_ESM.pdf]

Supplementary Information: ANXA11 biomolecular condensates facilitate protein-lipid phase coupling on lysosomal membranes

Jonathon Nixon-Abell[1]*, Francesco S. Ruggeri[2,3,4], Seema Qamar[1], Therese W. Herling[4], Magdalena A. Czekalska[4], Yi Shen[4,5,6], Guozhen Wang[1]†, Christopher King[7], Michael S. Fernandopulle[1,7,8], Tomas Sneideris[4], Joseph L. Watson[9], Visakh V.S. Pillai[2], William Meadows[1], James W. Henderson[1], Joseph E. Chambers[1], Jane L. Wagstaff[10], Sioned H. Williams[1], Helena Coyle[1], Greta Šneiderienė[4], Yuqian Lu[4], Shuyuan Zhang[4], Stefan J. Marciniak[1], Stefan M.V. Freund[10], Emmanuel Derivery[9], Michael E. Ward[7], Michele Vendruscolo[4], Tuomas P.J. Knowles[4], Peter St George-Hyslop[11,12]*

1. **Cambridge Institute for Medical Research, Department of Clinical Neurosciences, Clinical School, University of Cambridge, Cambridge, CB2 0XY, UK**
2. **Laboratory of Organic Chemistry, Wageningen University & Research, Stippeneng 4, 6703 WE, NL**
3. **Laboratory of Physical Chemistry and Soft matter, Wageningen University & Research, Stippeneng 4, 6703 WE, NL**
4. **Centre for Misfolding Diseases, Yusuf Hamied Department of Chemistry, University of Cambridge, Cambridge, CB2 1EW, UK**
5. **School of Chemical and Biomolecular Engineering, The University of Sydney, Sydney, NSW 2006, AU**
6. **The University of Sydney Nano Institute, The University of Sydney, Sydney, NSW 2006, AU**
7. **National institute for Neurological Disorder and Stroke, NIH, Bethesda, MD, USA**
8. **Medical Scientist Training Program, Feinberg School of Medicine, Northwestern University, Chicago, IL, USA**
9. **Cell Biology Division, MRC Laboratory of Molecular Biology, Cambridge, CB2 0QH, UK**
10. **NMR Facility, Structure Studies Division, MRC Laboratory of Molecular Biology, Francis Crick Avenue, Cambridge Biomedical Campus, Cambridge, CB2 0QH, UK**
11. **Department of Medicine (Division of Neurology), Temerty Faculty of Medicine, University Health Network, University of Toronto, Toronto, M5T 0S8, CA**
12. **Taub Institute for Research on Alzheimer's Disease and the Aging Brain, Department of Neurology, Columbia University Irvine Medical Center, 630 West 168th Street, New York, NY 10032, USA**

*Co-correspondence: ps2764@cumc.columbia.edu; jjn36@cam.ac.uk
†Present address: School of Medicine, Zhejiang University, Hangzhou, 310058, China.

Supplementary Figures

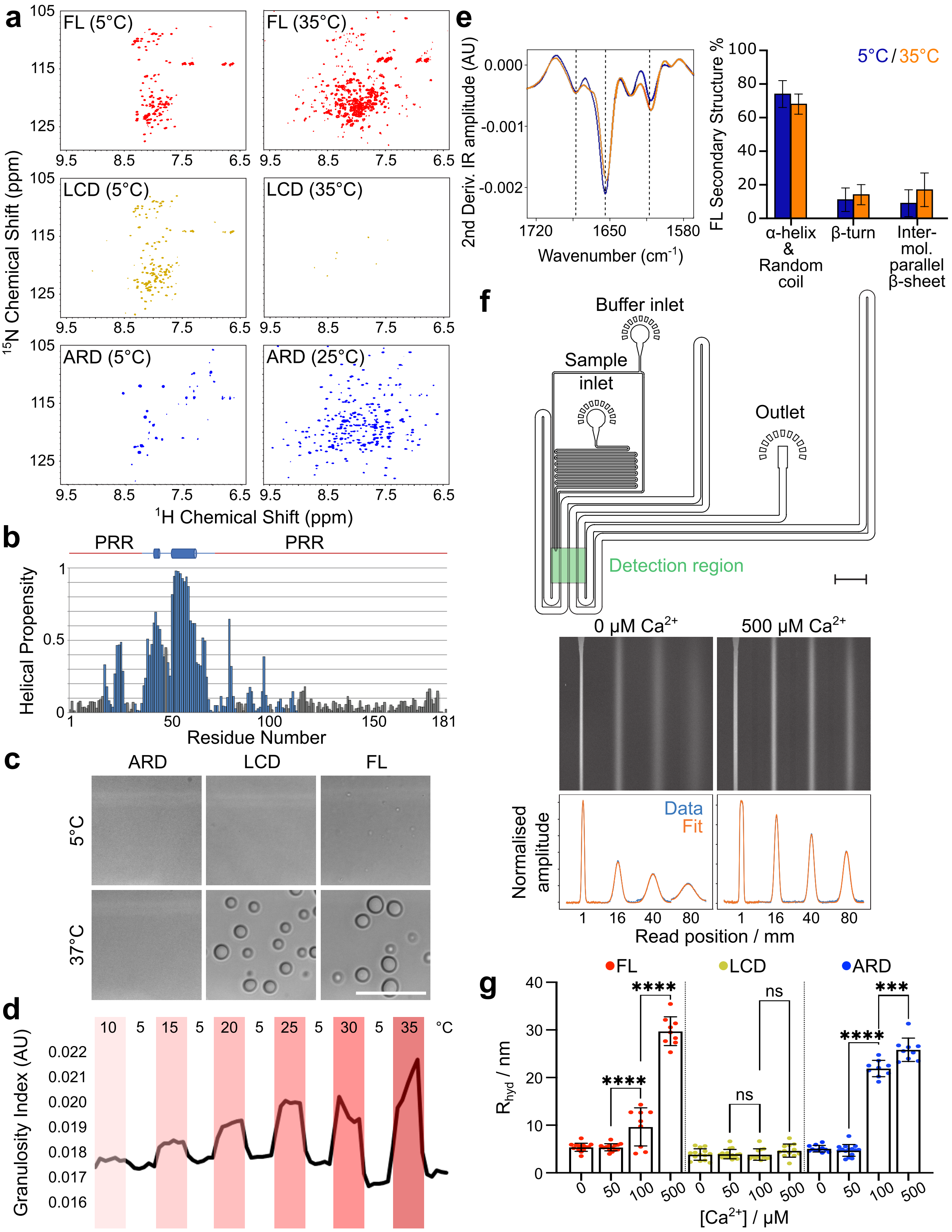

Supplementary Figure 1 - ANXA11 condensation and lipid membrane binding. (a) ^{1}H-^{15}N BEST-TROSY spectra of ANXA11 FL and LCD collected at 5°C and 35°C, and ARD collected at 5°C and 25°C. Each non-proline residue is represented by a peak in the spectrum, its location is indicative of the protein fold. A narrow ^{1}H distribution of peaks

characterizes disordered residues as observed for the LCD protein at 5°C, a wide ^{1}H distribution is seen for folded, globular proteins such as for the ARD at 25°C. These spectra illustrate the individual temperature dependency of the two domains. **(b)** Talos-N analysis based on the partial backbone assignment (blue residues, 53% of non-proline residues assigned) of LCD reveals two helical regions. Assignment of the two flanking, proline-rich regions (PRR) remains incomplete, however secondary structure predictions by Talos-N based on primary sequence are also reported (grey residues). **(c)** Bright-field images of 50 μM ANXA11 FL, LCD, and ARD imaged at 5 °C and 37 °C. Scale bar – 5 μm. Representative micrographs as seen across three independent experiments. **(d)** Quantification of 50 μM AF647 ANXA11 FL condensate formation during repeated cycles of temperature transitions. The granulosity index assesses the level of AXA11 condensation at any given temperature (see ANXA11 Temperature Cycling methods section). Recordings were taken across three independent experiments. **(e)** Second derivative FTIR spectra of the Amide I protein IR absorption region. Spectra were collected from 80 μM ANXA11 FL at 5 °C (dispersed state) and 35 °C (condensed state). The three dashed lines mark β-turn (left), α-helix and random coil (middle), and intermolecular parallel β-sheet (right) peak positions. On the right, a quantification of the % composition of ANXA11 secondary structure (Mean ± SD), demonstrating negligible changes between the dispersed and condensed state. Three spectra (256 co-averages) were collected across 5 experimental repeats. **(f)** Design of the microfluidic device for the diffusional sizing assay. Representative fluorescence micrographs show AF647-labelled ANXA11 FL at 0 and 500 μM Ca^{2+} incubated with SUVs as they pass through the microfluidic device. These data were analysed to obtain the diffusional profiles and fitted with numerical model simulations to obtain the hydrodynamic radius (see Microfluidic Diffusional Sizing Assays methods section). Scale bar – 1 mm. **(g)** Quantification of the hydrodynamic radius as established in (F) for SUVs incubated with 0.5 μM ANXA11 FL, LCD, or ARD at varying Ca^{2+} concentrations. Mean ± SD. One-way ANOVA with Tukey's multiple comparison, ***p = 0.0002, ****p < 0.0001, ns - not significant (p > 0.05), n=9 repeats (1.2-2.3x10^{8} SUVs).

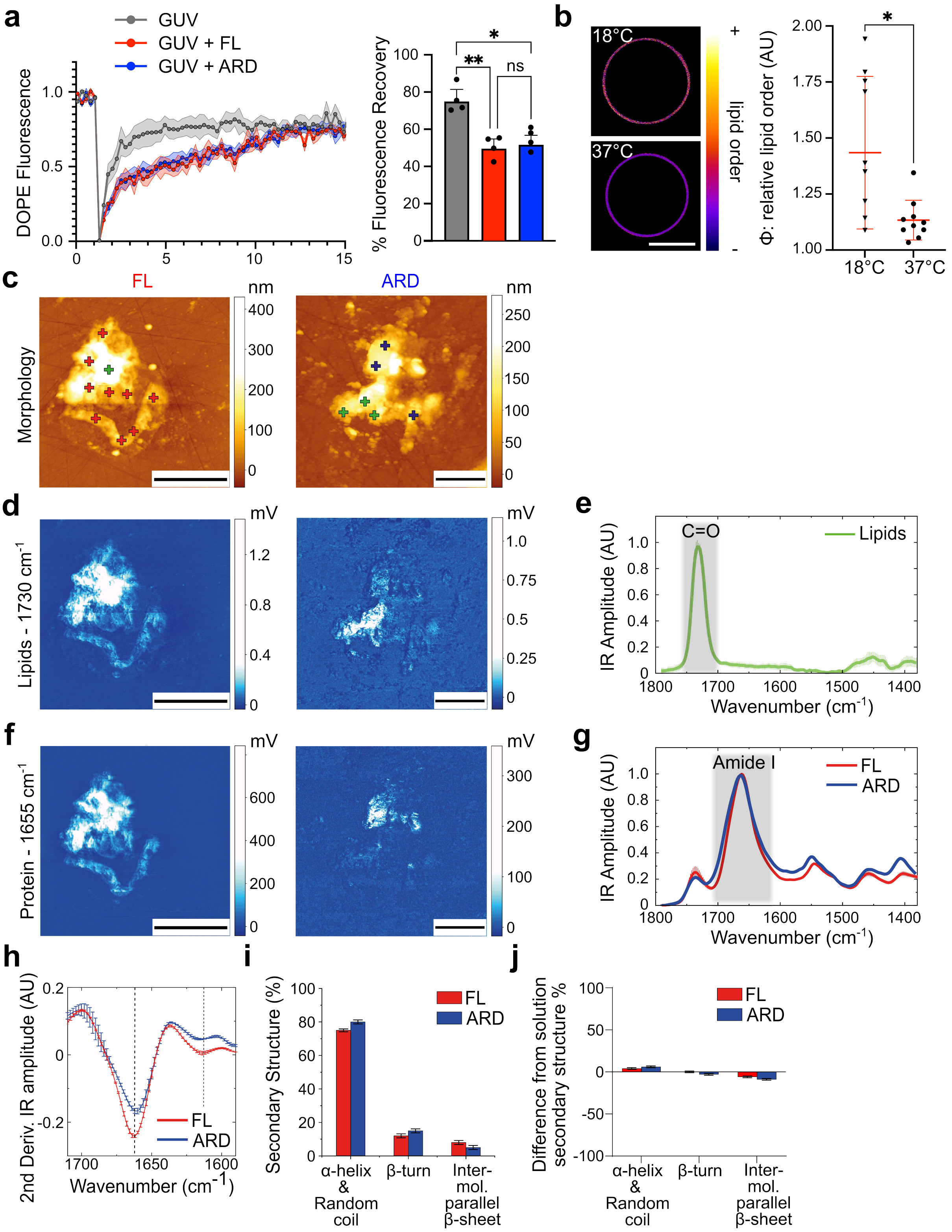

Supplementary Figure 2 – Chemical changes in protein and lipid components within ANXA11-GUV assemblies. (a) ATTO488 DOPE fluorescence recovery rates in GUVs in the presence of 100 μM Ca^{2+} co-incubated with either 0.5 μM ANXA11 FL or 0.5 μM ARD. The % fluorescence recovery by 5 sec is plotted alongside. Mean ± SD. One-way ANOVA with Tukey's multiple comparison, **p = 0.0039, *p = 0.0159, ns - not significant (p >

0.05), n=4 GUVs. **(b**) Fluorescence images of GUVs incubated with 5 μM PK dye to extract the relative order (ϕ) of membrane lipids at 18 °C and 37 °C. Corresponding quantification is plotted alongside. Mean ± SD. Unpaired two-tailed t-test with Welch's correction, *p = 0.0218, n=3 repeats (10 GUVs). **(c)** AFM-IR morphology maps of GUV fragments bound to 0.5 μM ANXA11 FL and 0.5 μM ARD at 100 μM Ca^{2+}. Scale bars - 5 μm (FL), 2 μm (ARD). N.B. the ARD panel shown here is used as the example in Figure 2G. **(d)** Nanoscale-resolved IR absorption maps collected at 1730 cm-1 (C=O stretching lipid region) of the ANXA11 FL and ARD GUVs described in (B). Scale bars - 5 μm (FL), 2 μm (ARD). **(e)** IR spectra (Mean ± SD, n=12 regions) collected from 'lipid only' regions (e.g. green crosses in (B)) show a maximum peak of absorption at 1730 cm^{-1} (C=O, lipids). **(f)** IR absorption maps collected at 1655 cm^{-1} (Amide I protein region) of the ANXA11 FL and ARD GUVs described in (B). Scale bars - 5 μm (FL), 2 μm (ARD). **(g)** IR spectra (Mean ± SD) collected from GUV fragments bound to ANXA11 FL (e.g. red crosses in (B), n=31 fragments) or ARD (e.g. blue crosses in (B), n=26) show a maximum peak of absorption at 1655 cm^{-1} (Amide I, protein). The SD in the ARD condition falls beneath the width of the line. **(h)** Second derivative IR spectra of the Amide I protein IR absorption region displayed in (F). The two dashed lines mark α-helix and random coil (left), and intermolecular parallel β-sheet (right) peak positions. **(i)** The secondary structural composition (Mean ± SD) derived from (G) of ANXA11-FL and ARD when bound to GUVs. **(j)** Difference-spectroscopy quantification of the deviation of the secondary structure (Mean ± SD) of ANXA11 FL and ARD bound to GUVs compared to the native secondary structure of the protein in solution (Supplementary Figure 1E). AFM-IR data reported in this figure was collected from across 69 independent GUV fragments (12 lipid only, 31 lipid+FL, 26 lipid+ARD) across 5 experimental repeats.

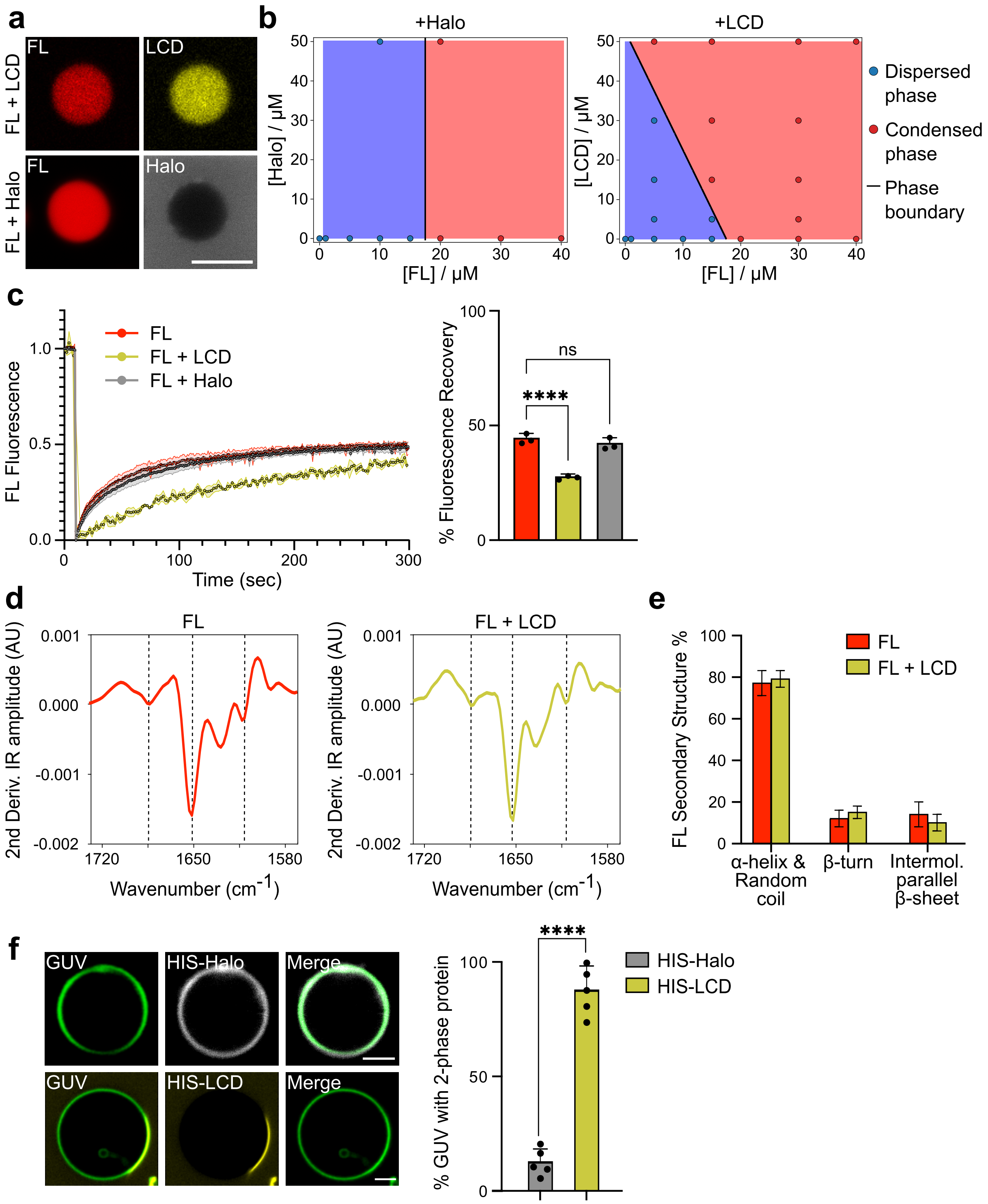

Supplementary Figure 3 – The impact of exogenous LCD-hemiprotein on ANXA11 phase state. (a) Fluorescence micrograph of individual AF647-ANXA11 FL condensates co-incubated with either AF488-LCD or JF549-Halo. Scale bar - 2.5 μm. Representative micrographs as seen across three independent experiments. **(b)** Phase diagrams plotting [ANXA11 FL] against either [Halo] or [LCD], with the phase boundary between the dispersed and condensed state marked with a black line. Each dot represents an imaging datapoint in which ANXA11 condensates were present (condensed phase), or not (dispersed phase) (see Generation of Phase Diagrams methods section). Data collected

across three independent experiments. **(c)** FRAP recovery curves of 25 μM recombinant AF647-ANXA11 FL alone or in the presence of unlabelled 25 μM LCD or 25 μM Halo. The corresponding quantification of the % fluorescence recovery at 150 sec is plotted alongside. Mean ± SD. One-way ANOVA with Dunnett's multiple comparison, ****p < 0.0001, ns - not significant (p > 0.05), n=3 condensates. N.B. The ANXA11 FL dataset displayed here is the same as in Supplementary Figure 4E as experiments were performed concurrently. **(d)** Second derivative FTIR spectra of the Amide I protein IR absorption region. Spectra collected are from 150 μM ANXA11 FL alone (left) or co-incubated with 150 μM LCD (right). The three dashed lines mark β-turn (left), α-helix and random coil (middle), and intermolecular parallel β-sheet (right) peak positions. **(e)** Quantification of the % composition of ANXA11 secondary structure (Mean ± SD). Note the low < 10% of intermolecular parallel beta sheet, even after addition of LCD. 19 spectra were collected across 5 experimental repeats. N.B. The FL dataset here is the same as in Supplementary Figure 4F as the experiments were conducted concurrently. **(f)** Representative fluorescence images of ATTO488 GUVs containing 2.5% NTA(Ni), incubated with either 25 μM HIS-LCD or HIS-Halo. Scale bar - 5 μm. On the right is the quantification (Mean ± SD) of % GUVs exhibiting dilute and dense phase protein co-existence. Unpaired two-tailed t-test with Welch's correction, ****p < 0.0001, n=5 repeats (32-48 GUVs).

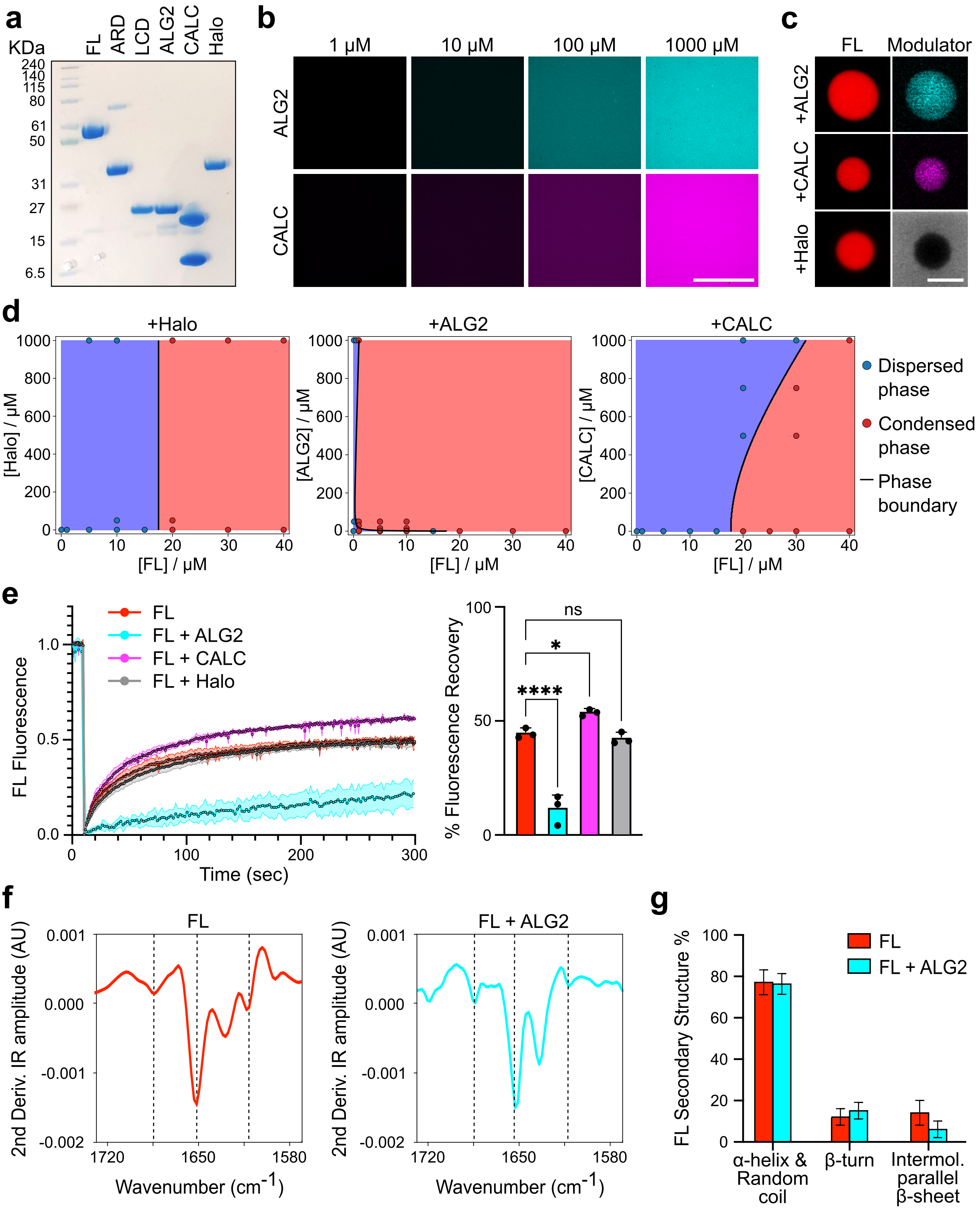

Supplementary Figure 4 – The impact of ALG2 and CALC on ANXA11 phase state. (a) Coomassie stained SDS-PAGE gel demonstrating the purity of recombinant proteins used throughout this study. **(b)** Fluorescence images of purified AF488-ALG2 and AF488-CALC at various protein concentrations. Note there are no visible condensates formed. 3 fields of view were examined in each condition with similar results. **(c)** Fluorescence micrograph of individual AF647-ANXA11 FL condensates co-incubated with either AF488-labelled ALG2, CALC, or Halo. Scale bar - 2.5 μm. 3 fields of view were examined in each condition with similar results. **(d)** Phase diagrams plotting [ANXA11 FL] against

either [ALG2] or [CALC], with the phase boundary between the dispersed and condensed state marked with a black line. Each dot represents an imaging datapoint in which ANXA11 condensates were present (condensed phase), or not (dispersed phase) (see Generation of Phase Diagrams methods section). Data collected across three independent experiments. **(e)** FRAP recovery curves of 25 μM recombinant AF647-ANXA11 FL alone or in the presence of unlabelled 0.1 mM ALG2, 1 mM CALC, or 1mM Halo. The corresponding quantification of the % fluorescence recovery at 150 sec is plotted alongside. Mean ± SD. One-way ANOVA with Dunnett's multiple comparison, *p = 0.0419, ****p < 0.0001, ns - not significant (p > 0.05), n=3 condensates. N.B. The ANXA11 FL dataset displayed here is the same as in Supplementary Figure 3C as experiments were performed concurrently. **(f)** Second derivative FTIR spectra of the Amide I protein IR absorption region. Spectra collected are from 150 μM ANXA11 FL alone (left) or coincubated with 600 μM ALG2 (right) (1[ANXA11]:4[ALG2]). The three dashed lines mark β-turn (left), α-helix and random coil (middle), and intermolecular parallel β-sheet (right) peak positions. **(g)** Quantification of the % composition of ANXA11 secondary structure (Mean ± SD). Note the low <10 % of intermolecular parallel beta sheet, even after addition of ALG2. 19 spectra were collected across 5 experimental repeats. N.B. The FL dataset here is the same as in Supplementary Figure 3E as the experiments were conducted concurrently.

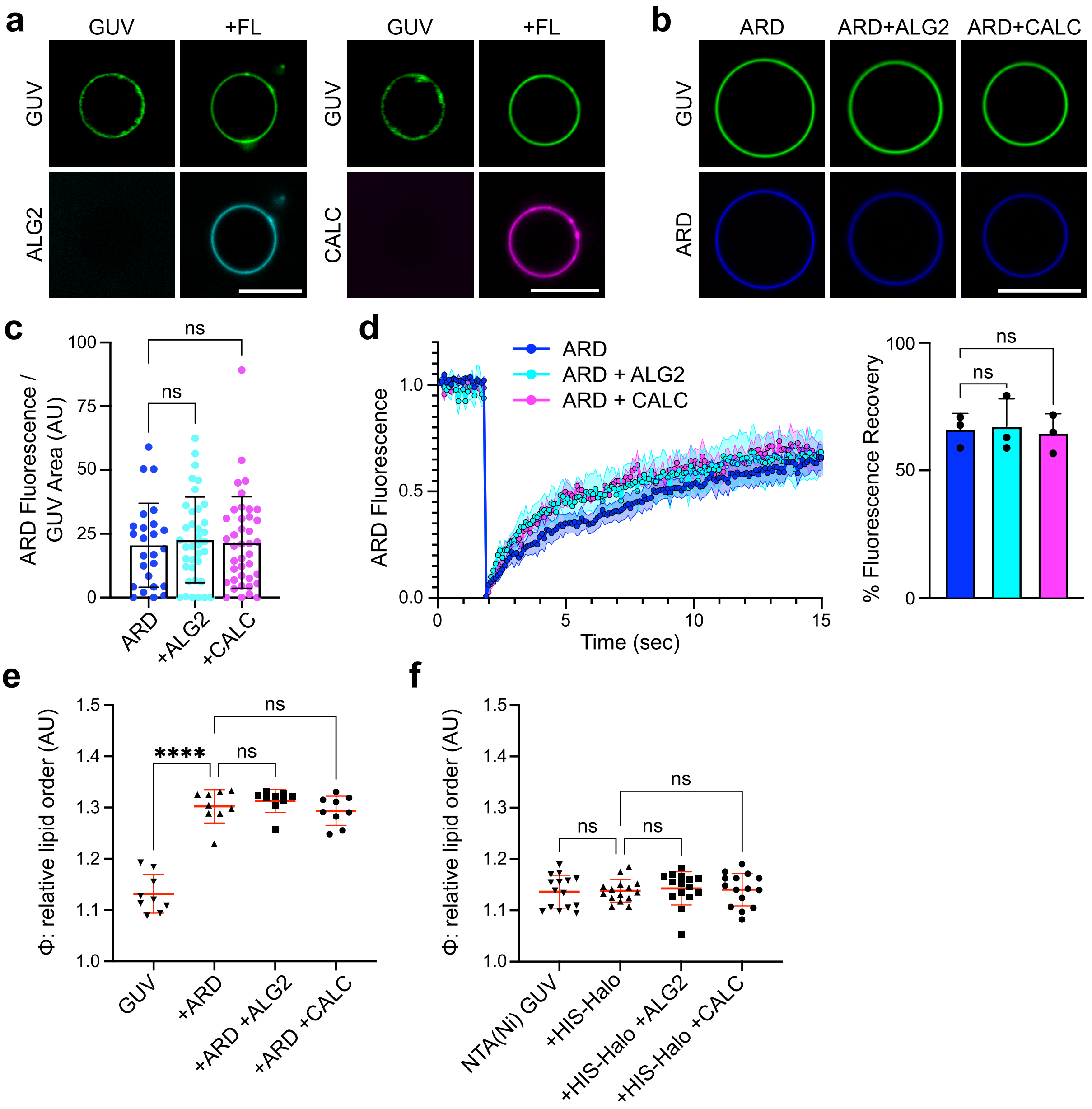

Supplementary Figure 5 – Effects of ALG2 and CALC on lipid membranes. (a) Fluorescence images of ATTO647 GUVs incubated with 100 µM Ca^{2+} and AF488-labelled 2 µM ALG2 or 20 µM CALC in the presence or absence of unlabelled 0.5 µM ANXA11 FL. Scale bar - 5 µm. At least 3 fields of view were assessed with similar results. **(b)** Representative fluorescence micrographs of ATTO488 GUVs incubated with 100 µM Ca^{2+} and 0.5 µM AF647-ARD, with and without unlabelled 2 µM ALG2 or 20 µM CALC. Scale bar – 5 µm. **(c)** Quantification of ARD recruitment to GUVs as displayed in (B). Mean ± SD. Kruskal-Wallis test with Dunn's multiple comparison, ns - not significant ($p > 0.05$) n=3 repeats (25-40 GUVs). **(d)** FRAP recovery curves of 0.5 µM AF647-ARD on the surface of GUVs in the presence or absence of 2 µM ALG2 or 20 µM CALC at 100 µM Ca^{2+}. The corresponding quantification of the % fluorescence recovery at 150 sec is plotted alongside. Mean ± SD. One-way ANOVA with Dunnett's multiple comparison, ns - not significant ($p > 0.05$), n=3 GUVs. N.B. The ARD condition is extracted from the same dataset used in Figure 3D. **(e)** Quantification of the relative lipid order (ϕ) of ATTO647 GUVs labelled with 5 µM PK dye incubated with 0.5 µM ARD and at 100 µM Ca^{2+} in the

presence or absence of 2 µM ALG2 or 20 µM CALC. Mean ± SD. One-way ANOVA with Tukey's multiple comparison, ****$p < 0.0001$, ns - not significant ($p > 0.05$) n=4 repeats (55-113 GUVs). **(f)** Quantification of the relative lipid order (ϕ) of NTA(Ni)-GUVs labelled with 5 µM PK dye. NTA(Ni) GUVs were incubated with 25 µM HIS-Halo and with either 0.1 mM ALG2 or 1 mM CALC. Mean ± SD. One-way ANOVA with Tukey's multiple comparison, ns - not significant ($p > 0.05$), n=5 repeats (17-64 GUVs).

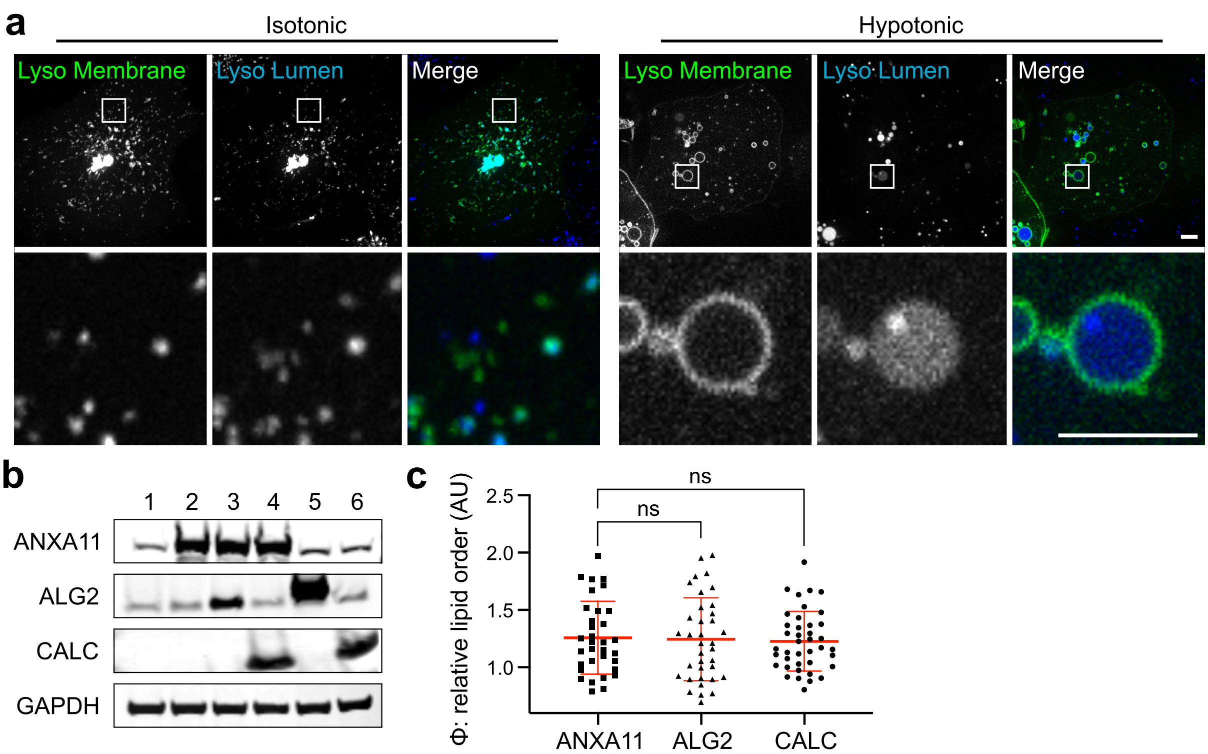

Supplementary Figure 6 – An *in cellula* system for studying the effects of ALG2 and CALC. (a) U2OS cells with fluorescently labelled lysosomal lumen (10-kDa dextran-AF594) and membrane (LAMP1-YFP) compartments, incubated in isotonic and hypotonic buffers (see Live Cell Microscopy PK dye Analysis methods section). The white ROI indicates zoomed regions displayed in the bottom row. Scale bars - 2 µm. Representative micrographs as seen across five independent experiments. **(b)** A Western blot of lysates from U2OS cells expressing the following constructs used to modulate ANXA11, ALG2 and CALC levels inside cells: (1) Untransfected; (2)ANXA11-T2A-TMEM192-Halo; (3)ANXA11-T2A-TMEM192-Halo/hPGK-ALG2; (4)ANXA11-T2A-TMEM192-Halo/hPGK-CALC; (5)ALG2-T2A-TMEM192-Halo; (6)CALC-T2A-TMEM192-Halo. Blots were probed with antibodies against ANXA11, ALG2, CALC and GAPDH. **(c)** Quantification of the relative lipid order (ϕ) of U2OS lysosomal membranes labelled with TMEM192-Halo-JF647 and incubated in 100 nM PK dye. Cells were expressing either ANXA11, or ALG2, or CALC. Mean ± SD. One-way ANOVA with Dunnett's multiple comparison, ns - not significant ($p > 0.05$), n=5 repeats (33-40 lysosomes).

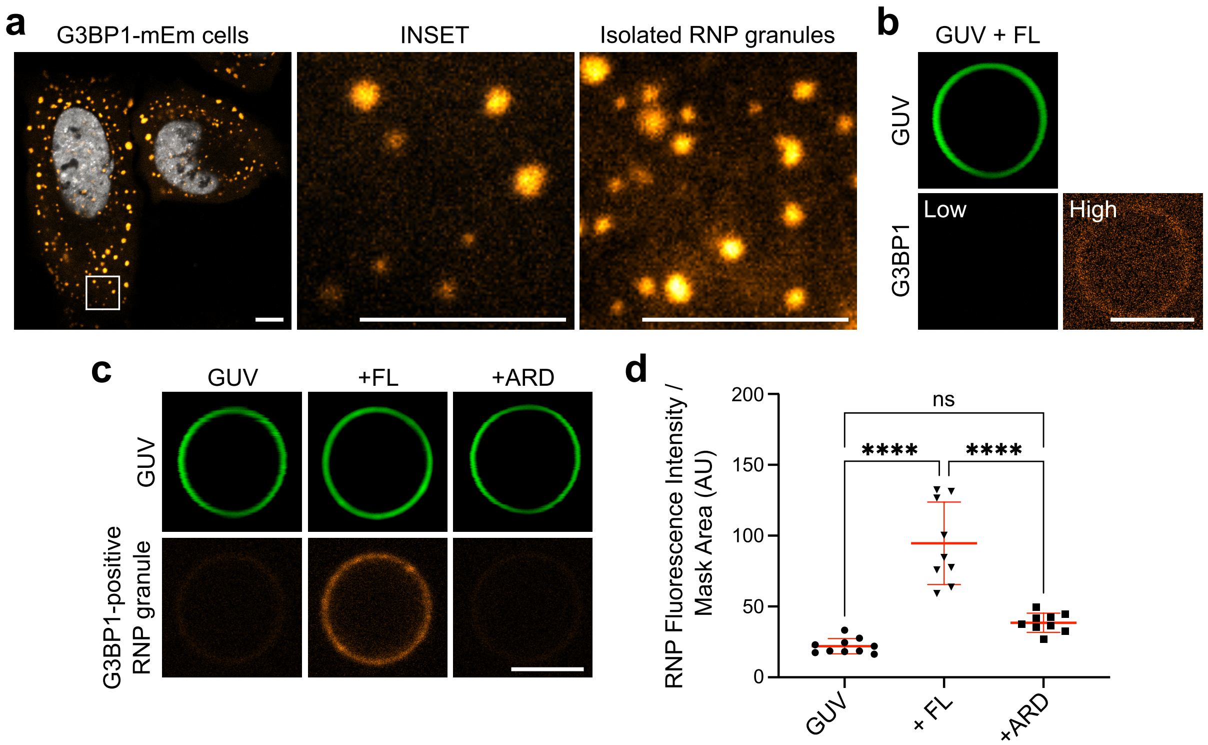

Supplementary Figure 7 – An *in vitro* RNP granule-ANXA11-GUV binding assay. (a) fluorescence images of a stable G3BP1-mEmerald (Orange) U2OS line labelled with after stress granule induction using 0.5 mM sodium arsenite for 30 min. SPY650-DNA was used to label the nucleus (white). On the right is an image of FAPS-isolated granules which have been pelleted and resuspended in a small volume of dilution buffer. Scale bar - 1.5 µm. 3 fields of view were examined with similar results. **(b)** An ATTO594 GUV incubated with 100 µM Ca^{2+} and 0.5 µM ANXA11 FL and 0.2 mg/ml of recombinantly purified mEmerald-G3BP1 at a low and high imaging exposure. Scale bar - 5 µm. 3 fields of view were examined with similar results. **(c)** Fluorescence images of ATTO594 GUVs incubated with 100 µM Ca^{2+} and either 0.5 µM ANXA11 FL or ARD. 0.2 mg/ml of G3BP1-mEmerald positive FAPS-isolated RNP granules were added to each condition. Scale bar - 5 µm. **(d)** Quantification of RNP granule recruitment to ANXA11-GUV assemblies as represented in (C). Mean ± SD. One-way ANOVA with Tukey's multiple comparison, ****p < 0.0001, ns - not significant (p > 0.05), n=3 repeats (40-106 GUVs).

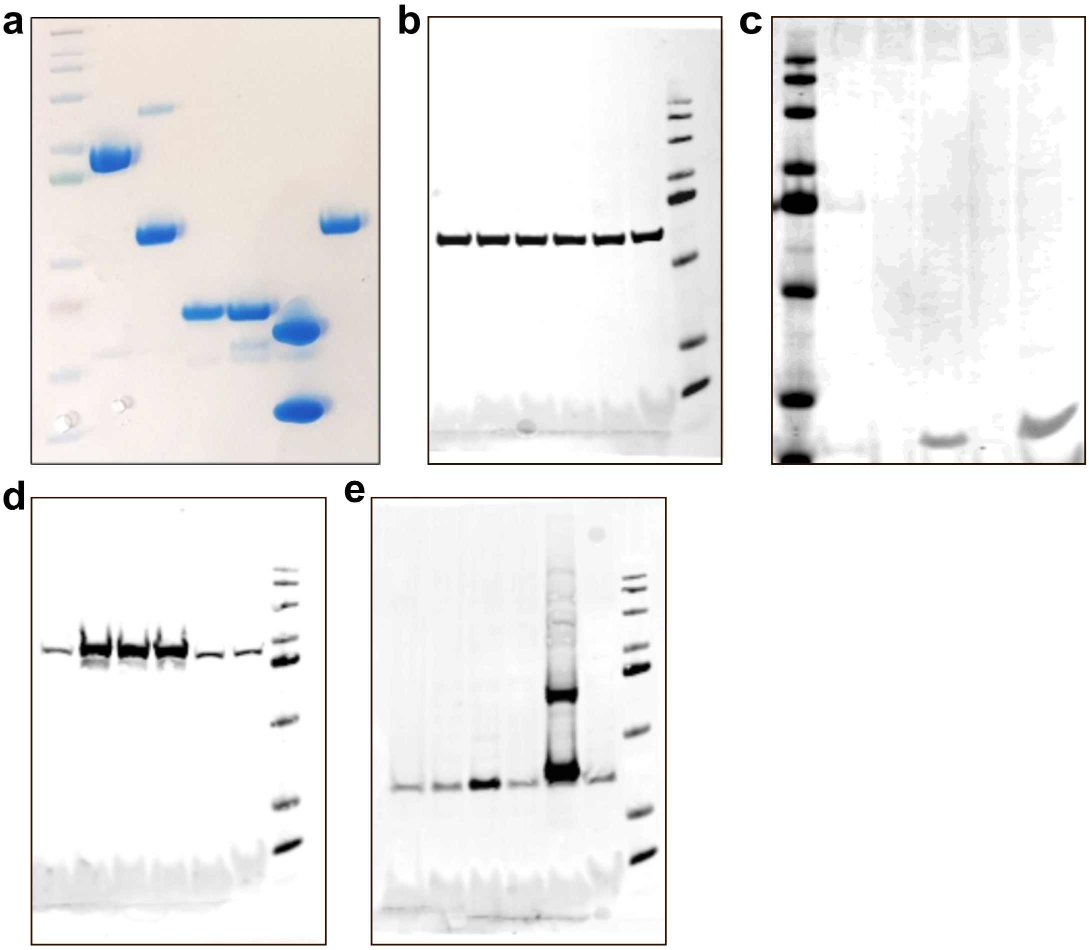

Supplementary Figure 8 – Uncropped blots and gels from manuscript. (a) Scan of uncropped SDS-PAGE gel from Supplementary Figure 4A. **(b-e)** Scan of uncropped Western blots as shown in Supplementary Figure 6B probed for GAPDH (b), Calcyclin (c), ANXA11 (d), ALG2 (e).

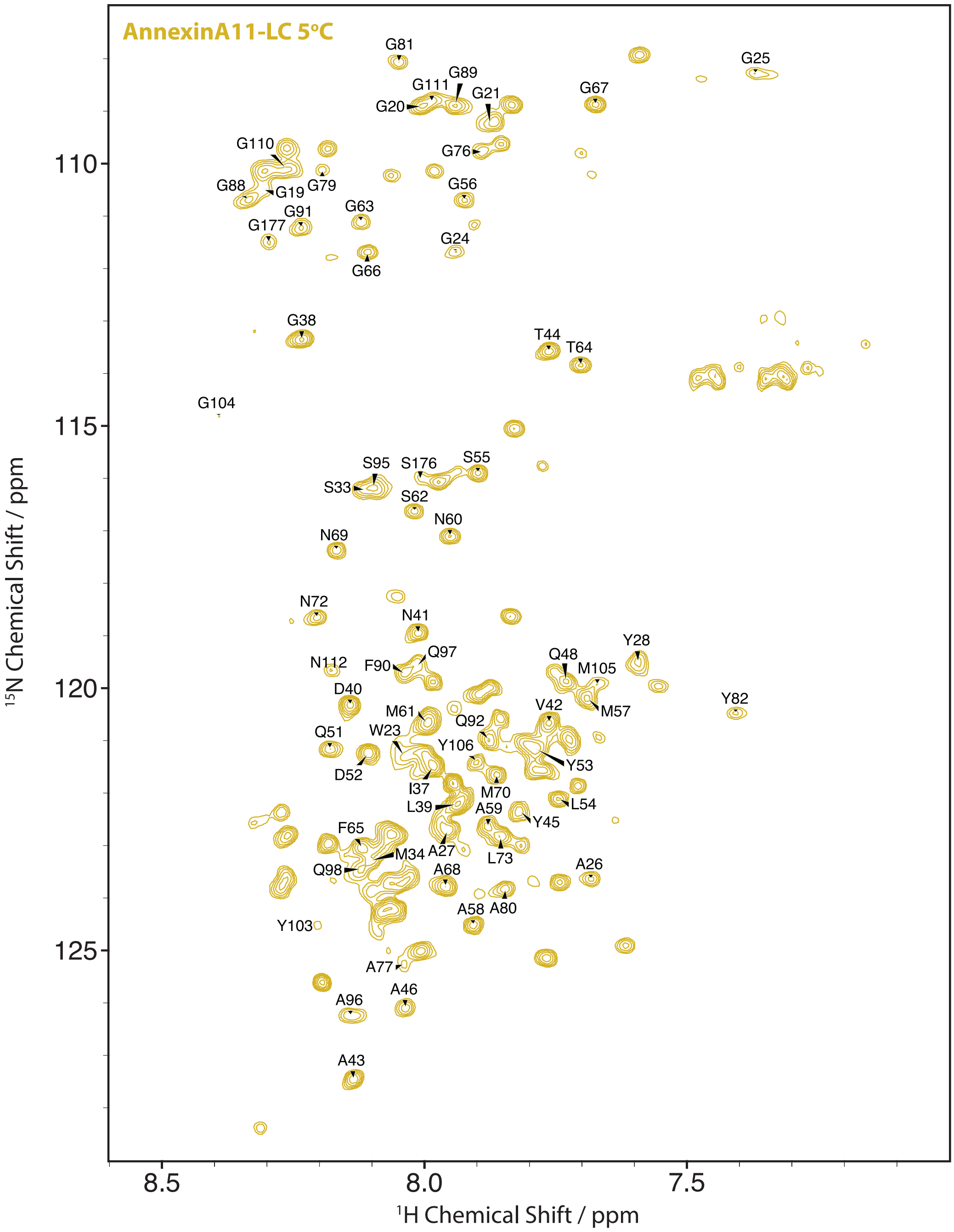

Supplementary Figure 9 – ^{1}H-^{15}N BEST-TROSY spectra of ^{15}N/^{13}C labelled ANXA11 LCD. Partial assignment of the Annexin A11 LCD enabled 68 of the 128 non-proline residues (53%) to be identified in the ^{1}H-^{15}N BEST-TROSY. Other non-assigned peaks belong to amino acids in the proline rich regions of the LCD.

Figure	Statistical test	n number	Plotted
1D	Kruskal-Wallis with Dunn's multiple comparison	3 (110-379 GUVs)	Mean ± SD
2B	One-way ANOVA with Tukey's multiple comparison	4 GUVs	Mean ± SD
2E	One-way ANOVA with Tukey's multiple comparison	3 (30-82 GUVs)	Mean ± SD
2I-K	One-way ANOVA with Tukey's multiple comparison	5 (12-31 GUV fragments)	Mean ± SD
3B	Kruskal-Wallis with Dunn's multiple comparison	3 (1171-1439 condensates)	Mean ± SD
3D	One-way ANOVA with Tukey's multiple comparison	4 GUVs	Mean ± SD
3F	Unpaired two-tailed t-test with Welch's correction	5 (44-126 GUVs)	Mean ± SD
3I	One-way ANOVA with Dunnett's multiple comparison	5 (83-114 GUVs)	Mean ± SD
4C	Kruskal-Wallis with Dunn's multiple comparison	3 (35-55 GUVs)	Mean ± SD
4D	Kruskal-Wallis with Dunn's multiple comparison	3 (117-281 GUVs)	Mean ± SD
4E	One-way ANOVA with Dunnett's multiple comparison	3-5 GUVs	Mean ± SD
4G	One-way ANOVA with Dunnett's multiple comparison	5 (58-134 GUVs)	Mean ± SD
4H	One-way ANOVA with Dunnett's multiple comparison	4 (28-94 GUVs)	Mean ± SD
5C	One-way ANOVA with Tukey's multiple comparison	4 (82-146 lysosomes)	Mean ± SD
6C	One-way ANOVA with Tukey's multiple comparison	3 GUVs	Mean ± SD
6F	One-way ANOVA with Tukey's multiple comparison	7 (21-78 GUVs)	Mean ± SD
S1G	One-way ANOVA with Tukey's multiple comparison	9 (1.2-2.3 x 10^8 SUVs)	Mean ± SD
S2A	One-way ANOVA with Tukey’s multiple comparison	4 GUVs	Mean ± SD
S2B	Unpaired two-tailed t-test with Welch's correction	3 (10 GUVs)	Mean ± SD
S3C	One-way ANOVA with Dunnett's multiple comparison	3 condensates	Mean ± SD
S3F	Unpaired two-tailed t-test with Welch's correction	5 (32-48 GUVs)	Mean ± SD
S4E	One-way ANOVA with Dunnett's multiple comparison	3 condensates	Mean ± SD
S5C	Kruskal-Wallis with Dunn's multiple comparison	3 (25-40 GUVs)	Mean ± SD
S5D	One-way ANOVA with Dunnett's multiple comparison	3 GUVs	Mean ± SD
S5E	One-way ANOVA with Tukey's multiple comparison	4 (55-113 GUVs)	Mean ± SD
S5F	One-way ANOVA with Tukey's multiple comparison	5 (17-64 GUVs)	Mean ± SD
S6C	One-way ANOVA with Dunnett's multiple comparison	5 (33-40 GUVs)	Mean ± SD
S7D	One-way ANOVA with Tukey's multiple comparison	3 (40-106 GUVs)	Mean ± SD

Supplementary Table 1 – Summary of statistical tests used throughout manuscript
