# Supplementary material for: ANXA11 biomolecular condensates facilitate protein-lipid phase coupling on lysosomal membranes
Source: Nat Commun. 2025 Mar 21;16:2814. doi: 10.1038/s41467-025-58142-5 (PMC11928461; doi:10.1038/s41467-025-58142-5)
Supplement: Supplementary file 2 — Description of Additional Supplementary Files [file 41467_2025_58142_MOESM2_ESM.pdf]

Description of Additional Supplementary Files

File Name: Supplementary Movie 1

Description: A timelapse confocal movie of 50µM AF647-ANXA11 FL incubated at 37oC in order to visualise condensate formation. A zoom panel is displayed inset. Scale bar – 10 µm.

File Name: Supplementary Data 1

Description: Proteins identified by mass spectrometry from FAPS-purified RNP granules (Figure 6) compared with a previously reported database from Youn et al., 2019.
