# Supplementary material for: ANXA11 biomolecular condensates facilitate protein-lipid phase coupling on lysosomal membranes
Source: Nat Commun. 2025 Mar 21;16:2814. doi: 10.1038/s41467-025-58142-5 (PMC11928461; doi:10.1038/s41467-025-58142-5)
Supplement: Supplementary file 5 — Reporting Summary [file 41467_2025_58142_MOESM5_ESM.pdf]

nature portfolio

Corresponding author(s): PSGH, JNA

Last updated by author(s): Jan 11, 2025

Reporting Summary

Nature Portfolio wishes to improve the reproducibility of the work that we publish. This form provides structure for consistency and transparency in reporting. For further information on Nature Portfolio policies, see our Editorial Policies and the Editorial Policy Checklist.

Statistics

For all statistical analyses, confirm that the following items are present in the figure legend, table legend, main text, or Methods section.

n/a	Confirmed	
☐	☒	The exact sample size (*n*) for each experimental group/condition, given as a discrete number and unit of measurement
☐	☒	A statement on whether measurements were taken from distinct samples or whether the same sample was measured repeatedly
☐	☒	The statistical test(s) used AND whether they are one- or two-sided<br>*Only common tests should be described solely by name; describe more complex techniques in the Methods section.*
☐	☒	A description of all covariates tested
☐	☒	A description of any assumptions or corrections, such as tests of normality and adjustment for multiple comparisons
☐	☒	A full description of the statistical parameters including central tendency (e.g. means) or other basic estimates (e.g. regression coefficient) AND variation (e.g. standard deviation) or associated estimates of uncertainty (e.g. confidence intervals)
☐	☒	For null hypothesis testing, the test statistic (e.g. *F*, *t*, *r*) with confidence intervals, effect sizes, degrees of freedom and *P* value noted<br>*Give P values as exact values whenever suitable.*
☒	☐	For Bayesian analysis, information on the choice of priors and Markov chain Monte Carlo settings
☒	☐	For hierarchical and complex designs, identification of the appropriate level for tests and full reporting of outcomes
☒	☐	Estimates of effect sizes (e.g. Cohen's *d*, Pearson's *r*), indicating how they were calculated

Our web collection on statistics for biologists contains articles on many of the points above.

Software and code

Policy information about availability of computer code

Data collection	ZEN Black v2.3 (Zeiss)
Data analysis	FIJI v1.54d (NIH), PRISM 9 (Graphpad), OriginPRO v10.05 (OriginLab),

For manuscripts utilizing custom algorithms or software that are central to the research but not yet described in published literature, software must be made available to editors and reviewers. We strongly encourage code deposition in a community repository (e.g. GitHub). See the Nature Portfolio guidelines for submitting code & software for further information.

Data

Policy information about availability of data

All manuscripts must include a data availability statement. This statement should provide the following information, where applicable:

- Accession codes, unique identifiers, or web links for publicly available datasets
- A description of any restrictions on data availability
- For clinical datasets or third party data, please ensure that the statement adheres to our policy

The authors declare that data supporting the findings of this study are available within the paper and its supplementary information files. A source data file is provided with the manuscript and raw data including uncropped blots are available on Figshare (DOI: 10.6084/m9.figshare.25746051).

Research involving human participants, their data, or biological material

Policy information about studies with human participants or human data. See also policy information about sex, gender (identity/presentation), and sexual orientation and race, ethnicity and racism.

Reporting on sex and gender	N/A
Reporting on race, ethnicity, or other socially relevant groupings	N/A
Population characteristics	N/A
Recruitment	N/A
Ethics oversight	N/A

Note that full information on the approval of the study protocol must also be provided in the manuscript.

Field-specific reporting

Please select the one below that is the best fit for your research. If you are not sure, read the appropriate sections before making your selection.

- [x] Life sciences
- [ ] Behavioural & social sciences
- [ ] Ecological, evolutionary & environmental sciences

For a reference copy of the document with all sections, see nature.com/documents/nr-reporting-summary-flat.pdf

Life sciences study design

All studies must disclose on these points even when the disclosure is negative.

Sample size	Sample sizes for experiments are clearly stated throughout the manuscript. The n number of experiments is listed alongside the total number of cells/GUVs analysed per experiment. Sample sizes were selected based on variance observed in prior experiments of a similar nature and to meet appropriate requirements to permit statistical interrogation of the data.
Data exclusions	No data were excluded from the analyses
Replication	All replicates are included in the manuscript
Randomization	No formal randomization method was required for these types of data.
Blinding	Investigators were not blinded to the experimental group allocation as the analysis methods employed within this study are not influenced by the group allocation.

Reporting for specific materials, systems and methods

We require information from authors about some types of materials, experimental systems and methods used in many studies. Here, indicate whether each material, system or method listed is relevant to your study. If you are not sure if a list item applies to your research, read the appropriate section before selecting a response.

Materials & experimental systems

n/a	Involved in the study	
[ ]	[x]	Antibodies
[ ]	[x]	Eukaryotic cell lines
[x]	[ ]	Palaeontology and archaeology
[x]	[ ]	Animals and other organisms
[x]	[ ]	Clinical data
[x]	[ ]	Dual use research of concern
[x]	[ ]	Plants

Methods

n/a	Involved in the study	
[x]	[ ]	ChIP-seq
[x]	[ ]	Flow cytometry
[x]	[ ]	MRI-based neuroimaging

Antibodies

Antibodies used	ANXA11 Rab polyclonal, Proteintech, catalog #:10479-2-AP<br>ALG2 Rab monoclonal (clone: EPR3260), Abcam, catalog #:ab133326<br>CALC Rab polyclonal, Proteintech, catalog #:10245-1-AP

	GAPDH Rab monoclonal (clone: D16H11), Cell Signalling, catalog #:5174S
Validation	ANXA11 Rab polyclonal – validated by Western Blot in ANXA11 KO human cells (HeLa)<br>ALG2 Rab monoclonal – specific single band at correct molecular weight in multiple human cell lines by Western Blot (HeLa, Jurkat, HepG2)<br>CALC Rab polyclonal – validated by Western Blot in siRNA ANXA11 KD in human cells (HeLa)<br>GAPDH Rab monoclonal – specific single band at correct molecular weight in multiple human cell lines by Western Blot (HeLa, NIH/3T3, C6, COS7)

Eukaryotic cell lines

Policy information about cell lines and Sex and Gender in Research

Cell line source(s)	U-2 OS (U2OS in the manuscript) cells were acquired from ATCC (HTB-96). The U-2 OS cell line was derived from a 15-year-old, White, female osteosarcoma patient.
Authentication	Authentication details are listed on the ATCC webpage (https://www.atcc.org/products/htb-96#detailed-product-information)
Mycoplasma contamination	This cell line was tested to be negative for mycoplasma
Commonly misidentified lines<br>(See ICLAC register)	N/A
